# Association between frailty and echocardiographic findings in hospitalized older adults with preserved ejection fraction

**DOI:** 10.1093/ehjopen/oeaf087

**Published:** 2025-07-04

**Authors:** Daniel Betancourt, Jose Zuluaga, Fernando Arango, Tatiana Murillo, Daniel Hincapié

**Affiliations:** Research Group on Geriatrics and Gerontology, Faculty of Health Sciences, Universidad de Caldas, Carrera 25 # 48-57, Manizales 170004, Colombia; Department of Cardiology, SES Hospital Universitario de Caldas, Universidad de Manizales, Calle 72 # 25-75, Manizales 170004, Colombia; Master of Science in Epidemiology, SES Hospital Universitario de Caldas, Universidad de Manizales, Carrera 9A # 10-03, Manizales 170001, Colombia; Clinical Department, Faculty of Health Sciences, Universidad de Caldas, Intensive Care Unit, Hospital Universitario Santa Sofía, Carrera 25 # 55A-06, Manizales 170001, Colombia; Emergency Medicine Section, Universidad de Antioquia, Calle 70 # 10-03, Medellín 050010, Colombia

**Keywords:** Frailty, Diastolic dysfunction, Older adults, Preserved ejection fraction, Echocardiography

## Abstract

**Aims:**

This study aims to examine the association between frailty and cardiac structure and function in hospitalized older adults with preserved ejection fraction, using echocardiographic parameters and the Fried frailty index.

**Methods and results:**

A cross-sectional analytical study was conducted in two referral centres. A total of 269 individuals aged 60 years or older were included. The exclusion criteria were conditions that affect ventricular mechanics. Patients were categorized into non-frail, prefrail, and frail groups. Transthoracic echocardiography included 2D imaging, Doppler, and Global Longitudinal Strain (GLS) of the left ventricle. Comparative analysis was considered statistically significant if *P* < 0.05. Frailty was significantly associated with diastolic dysfunction, with an adjusted odds ratio of 3.49 (95% CI: 1.90–6.39, *P* < 0.001). After adjusting for potential confounders—including age, hypertension, diabetes mellitus, coronary heart disease, chronic obstructive pulmonary disease, and chronic kidney disease—frailty remained strongly associated with diastolic dysfunction. In addition, frail patients exhibited distinctive cardiac structural changes, including larger atrial volumes and smaller ventricular volumes. Pulmonary artery systolic pressure and tricuspid regurgitation velocity were also significantly elevated in frail individuals, while GLS of the left ventricle did not differ between groups.

**Conclusion:**

Frailty is independently associated with diastolic dysfunction. Even after adjusting for key comorbidities, it remains strongly associated with significant structural and functional cardiac alterations in hospitalized older adults with preserved ejection fraction.

## Structured Graphical Abstract

**Graphical Abstract:**
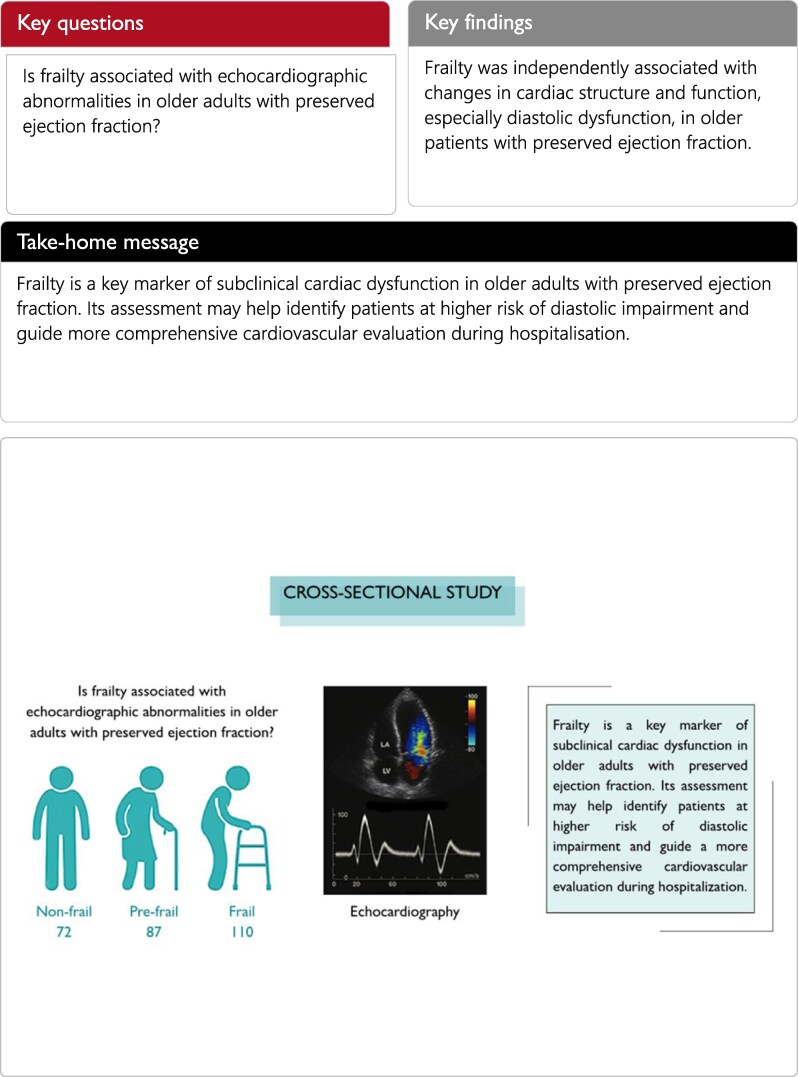

## Introduction

Since 1980, for the first time in history, most people can expect to live beyond the age of 60 years.^1^ However, as life expectancy continues to increase worldwide, there has also been a rise in the prevalence of non-communicable chronic diseases, particularly cardiovascular diseases such as heart failure.^2^ Moreover, geriatric syndromes such as frailty have emerged, defined as a biological syndrome characterized by a progressive decline in physiological reserves and resistance to stressors due to the cumulative dysfunction of multiple physiological systems, leading to increased vulnerability to adverse outcomes.^3–5^ This condition not only has health, social, and economic implications but also represents a significant challenge in public health.^6^

The relationship between frailty and cardiovascular disease has been widely studied,^7^ but whether frailty itself leads to worse clinical outcomes by directly affecting cardiac structure and function remains controversial.^8^ Echocardiography has evolved as a key tool in cardiovascular assessment, with the quantification of chamber size and function being the cornerstone of cardiac imaging.^8^ Some studies have suggested an association between frailty and increased left ventricular mass,^9–11^ whereas others have not supported this conclusion.^12–14^ Furthermore, previous research has failed to demonstrate a significantly lower left ventricular ejection fraction in frail individuals.^9,12,15^

The aim of this study was to assess frailty using the physical phenotype derived from the Cardiovascular Health Study^16^ in an older population hospitalized in two referral centres and to analyse its association with cardiac structure and function.

## Methods

### Study population

A total of 269 older individuals aged 60 and above, who walk independently or with assistive devices such as a cane or walker, and who underwent a clinically indicated transthoracic echocardiogram at SES Hospital Universitario de Caldas and Hospital Universitario Santa Sofía in Manizales, Colombia, from July 2023 to June 2024, were prospectively enrolled.

The exclusion criteria were patients with congenital heart disease, cardiomyopathy, moderate or severe valvular heart disease, severe right ventricular dilation with septal displacement and dyssynchrony, segmental wall motion abnormalities, valvular prostheses or cardiac devices, immunochemotherapy, delirium, hospitalized in the intensive care unit, poor acoustic window, left ventricular ejection fraction < 50%, and acute heart failure at the time of admission. Acute heart failure was defined as the presence of typical symptoms (e.g. dyspnoea, orthopnoea, and oedema) along with elevated NT-proBNP levels (> 900 pg/mL for patients ≤ 75 years and > 1800 pg/mL for those > 75 years).

This study was conducted in accordance with the Declaration of Helsinki, and participants were required to provide written informed consent. The study protocol was approved by the ethics committees of each participating hospital (approval numbers: R-20230421-1 and DEI-054-23).

### Definition of frailty

The frailty phenotype was assessed using the Fried Frailty Index.^16^ The criteria for defining frailty included weight loss, exhaustion, physical activity, gait speed, and grip strength.

- Weight loss was defined as an unintentional loss of more than 10 pounds (4.5 kg) in the previous year.
- Exhaustion was determined based on self-reported responses to two items from the Centre for Epidemiologic Studies Depression Scale.^17^
- Physical activity was assessed using the exercise scale to measure function in advanced activities of daily living.
- Grip strength values were obtained using a Takei Smedley Hand Dynamometer III, which measures grip strength in kilograms-force (kg/f). Weakness was defined as values below 26 kg/f for men and below 16 kg/f for women, based on the cut-off points established by the Foundation for the National Institutes of Health.^18^
- Gait speed, measured in metres per second (m/s), is a validated parameter in the literature, typically assessed over distances of 3, 5, or 10 m.^19^ In this study, the 3-m assessment was used. Each patient underwent two gait speed measurements, and the highest recorded speed was used for analysis. Slowness was defined as a gait speed below 0.8 m/s, based on the cut-off point established in the International Mobility in Ageing Study.^20^

Patients were classified as ‘frail’ if they had impairments in ≥ 3 components, as ‘prefrail’ if they had impairments in 1 or 2 components, and as ‘non-frail’ if they had no apparent impairments.

### Collection of data for clinical variables

The clinical information, including demographic data, medical history, and structural and functional echocardiographic variables, was obtained from medical records. The variables used to define frailty were assessed by two of the authors, who are experts in geriatrics.

### Echocardiography

Echocardiographic images of the participants were obtained using a Philips EPIQ CVX echocardiograph, available at both institutions. Standard 2D views of three to five cardiac cycles were collected: three cycles for patients with sinus rhythm and five cycles for those with atrial fibrillation, following guideline recommendations.^21^ Cardiac structure and function were assessed using two-dimensional transthoracic echocardiography, colour Doppler, and spectral Doppler. Additionally, the Global Longitudinal Strain (GLS) of the left ventricle was analysed in all patients.

Diastolic dysfunction was defined according to current literature,^22^ using the following criteria: (i) average *E/e*′ ratio > 14; (ii) *e*′ velocity: < 7 cm/s septal or < 10 cm/s lateral; (iii) tricuspid regurgitation velocity > 2.8 m/s; and (iv) left atrial volume index > 34 mL/m². Diastolic dysfunction was diagnosed when more than 50% of the specified criteria were met.

All transthoracic echocardiograms in this study were performed and interpreted by a single echocardiography specialist with Level 3 training. Frailty was assessed independently by the study authors using Fried’s criteria, and they were blinded to the echocardiographic findings at the time of data collection.

### Statistical analyses

According to the Fried phenotype score, patients were classified into three groups: non-frail, prefrail, and frail.

Continuous variables were expressed as means ± standard deviation if they followed a normal distribution, as determined by the Kolmogorov–Smirnov test. If they did not follow a normal distribution, they were expressed as median and inter-quartile range. Differences between groups were compared using one-way ANOVA. Categorical variables were expressed as counts and frequency (%), and comparisons between groups were made using the χ^2^ test or Fisher’s exact probability method, as appropriate.

Logistic regression models were used to analyse the association between clinical data, structural and functional cardiac parameters, and frailty in older adults. Odds ratios (ORs) and 95% confidence intervals (CIs) were reported. A *P*-value of < 0.05 was considered statistically significant. All statistical analyses were performed using Stata software, version 16.1.

## Results

### Baseline characteristics

A total of 269 hospitalized patients were included, with a range between 60 and 96 years and a mean of 74.5 ± 8.9 years (95% CI: 73.4–75.6). The gender distribution was 54.4% female and 45.6% male. According to the Fried phenotype, 40.9% of participants were classified as frail, 32.3% as prefrail, and 26.8% as non-frail.

Clinical characteristics showed a high prevalence of hypertension (66.5%), followed by diabetes mellitus (26.0%) and chronic obstructive pulmonary disease (COPD) (21.6%). Additionally, chronic kidney disease (9.7%), atrial fibrillation (6.3%), and coronary artery disease (10.4%) were reported. *Table 1* presents the baseline characteristics of the study population.

**Table 1:** Baseline characteristics of non-frail, prefrail, and frail patients

	Non-frail (*n* = 72)	Prefrail (*n* = 87)	Frail (*n* = 110)	*P*
Male	42 (58.3%)	43 (49.4%)	38 (34.6%)	**0**.001*
Age (years)	68.4 ± 6.2	75.1 ± 8.3	78.1 ± 8.9	**<0**.**001**^†^
Age >80 (years)	3 (4.2%)	24 (27.6%)	52 (47.3%)	**<0**.**001**^‡^
BMI (kg/m^2^)	26.0 ± 3.7	24.9 ± 4.0	24.8 ± 4.6	0.088^†^
Arterial hypertension	43 (59.7%)	60 (69.0%)	76 (69.1%)	0.358*
Diabetes mellitus	11 (15.3%)	30 (34.5%)	29 (26.4%)	**0**.023*
Atrial fibrillation	3 (4.2%)	5 (5.8%)	9 (8.2%)	0.567^‡^
CKD	7 (9.7%)	8 (9.2%)	11 (10.0%)	1.000^‡^
COPD	3 (4.2%)	16 (18.4%)	39 (35.5%)	**<0**.**001**^‡^
OSA	3 (4.2%)	1 (1.2%)	4 (3.6%)	0.436^‡^
Coronary heart disease	9 (12.5%)	10 (11.5%)	9 (8.2%)	0.597^‡^
Smoking history	5 (6.9%)	5 (5.8%)	8 (7.3%)	0.909^‡^
NYHA ≥ III	3 (4.2%)	13 (14.9%)	53 (48.2%)	**<0**.**001**^‡^

BMI, body mass index; CKD, chronic kidney disease; COPD, chronic obstructive pulmonary disease; OSA, obstructive sleep apnoea; NYHA, New York Heart Association functional class.

Values shown in bold indicate statistically significant differences (*P* < 0.05) observed when comparing the results across the three groups: non-frail, pre-frail, and frail patients.

^*^χ^2^ test.

^†^Analysis of variance test (ANOVA).

^‡^Fisher’s exact test.

### Echocardiographic structural parameters

Frail patients had smaller left ventricular indexed volumes (LVEDVI: 53.3 vs. 55.6 mL/m^2^ in prefrail and 54.6 mL/m^2^ in non-frail, *P* = 0.007) and larger left atrial indexed volumes (LAVI: 39.6 vs. 35.0 mL/m^2^ in prefrail and 30.5 mL/m^2^ in non-frail, *P* = 0.019). A progressive increase in relative wall thickness (RWT) was also observed as frailty severity increased (0.38 in non-frail vs. 0.40 in prefrail and 0.42 in frail, *P* = 0.043). *Table 2* lists the cardiac structural parameters.

**Table 2:** Cardiac structural parameters of non-frail, prefrail, and frail patients

	Non-frail (*n* = 72)	Prefrail (*n* = 87)	Frail (*n* = 110)	P*
LVESD (mm)	25.7 ± 4.0	26.1 ± 4.8	25.0 ± 4.7	0.603
LVEDD (mm)	43.3 ± 3.9	41.9 ± 4.9	41.3 ± 6.3	**<0**.**001**
IVS (mm)	8.3 ± 1.3	8.5 ± 1.4	8.8 ± 1.6	0.132
LVPW (mm)	8.1 ± 1.2	8.2 ± 1.2	8.7 ± 1.8	0.349
LVEDV (mL/m^2^)	54.6 ± 10.4	55.6 ± 9.5	53.3 ± 12.6	**0**.**007**
LVESV (mL/m^2^)	22.9 ± 6.7	22.5 ± 5.0	21.8 ± 5.8	**0**.**006**
RWT	0.38 ± 0.06	0.40 ± 0.07	0.42 ± 0.07	**0**.**043**
LVMI (g/m^2^)	63.1 ± 14.0	66.0 ± 14.5	73.0 ± 22.1	0.286
RVD_basal_ (mm)	35.7 ± 4.8	36.5 ± 5.4	36.7 ± 5.6	0.901
RVD_mid_ (mm)	31.6 ± 4.5	32.0 ± 4.5	32.5 ± 5.0	0.625
RVD_long_ (mm)	68.9 ± 7.4	67.5 ± 7.6	67.7 ± 7.4	0.096
LAD (mm)	39.1 ± 5.0	39.5 ± 7.7	40.7 ± 6.9	0.493
LAVI (mL/m^2^)	30.5 ± 8.0	35.0 ± 8.8	39.6 ± 13.3	**0**.**019**
RAVI (mL/m^2^)	21.5 ± 6.7	23.9 ± 6.6	26.0 ± 9.1	**0**.**015**

LVESD, left ventricular end systolic diameter; LVEDD, left ventricular end diastolic diameter; IVS, interventricular septum; LVPW, left ventricular posterior wall; LVEDV, left ventricular end diastolic volume; LVESV, left ventricular end systolic volume; RWT, relative wall thickness; LVMI, left ventricular mass index; RVD_basal_, right ventricular basal diameter; RVD_mid_, right ventricular mid diameter; RVL_long_, right ventricular longitudinal diameter; LAD, left atrial diameter; LAVI, left atrial volume index; RAVI, right atrial volume index.

Values shown in bold indicate statistically significant differences (*P* < 0.05) observed when comparing the results across the three groups: non-frail, pre-frail, and frail patients.

^*^Analysis of variance (ANOVA) test.

*LVMI non-frail* vs. *frail P*: 0.000 (Student’s *t*-test).

### Echocardiographic functional parameters

In terms of functional parameters, no significant differences were found in left ventricular ejection fraction (LVEF) among the groups (*P* = 0.784). However, frail patients exhibited significant diastolic abnormalities, reflected by a progressive decrease in the *E/A* ratio (0.83 in non-frail vs. 0.77 in frail, *P* = 0.014) and an increase in the *E/e*′ ratio (8.7 ± 2.4 in non-frail vs. 12.4 ± 6.4 in frail, *P* = 0.003).

Additionally, pulmonary artery systolic pressure (PASP) was significantly higher in frail patients (41.0 vs. 36.7 mmHg in non-frail, *P* = 0.002). *Table 3* presents the cardiac functional parameters.

**Table 3:** Cardiac functional parameters of non-frail, prefrail, and frail patients

	Non-frail (*n* = 72)	Prefrail (*n* = 87)	Frail (*n* = 110)	P*
LVEF (%)	59.6 ± 3.7	58.8 ± 4.6	59.7 ± 3.9	0.784
*E/A*	0.83 ± 0.25	0.80 ± 0.26	0.77 ± 0.94	**0**.**014**
*E/e*′	8.7 ± 2.4	10.6 ± 3.6	12.4 ± 6.4	**0**.**003**
*Se′V* (cm/s)	6.2 ± 1.4	5.6 ± 1.4	5.0 ± 1.4	**<0**.**001**
*Le*′*V* (cm/s)	7.9 ± 1.7	7.0 ± 2.1	6.5 ± 1.9	**<0**.**001**
TRV (m/s)	2.6 ± 0.3	2.9 ± 0.3	2.9 ± 0.5	**0**.**004**
PASP (mmHg)	36.7 ± 10.2	39.7 ± 12.8	41.0 ± 11.0	**0**.**002**
TAPSE (mm)	21.7 ± 2.5	21.5 ± 3.2	21.7 ± 3.8	0.759
TASA (cm/s)	12.2 ± 2.1	12.7 ± 2.7	12.3 ± 2.9	0.601
FAC (%)	48.2 ± 6.1	48.6 ± 6.9	48.0 ± 5.8	0.531
LVGLS (%)	−23.0 ± 2.0	−22.0 ± 2.6	−21.9 ± 2.7	0.592

LVEF, left ventricular ejection fraction; *Se′V*, septal *e*′ velocity; *Le*′*V*, lateral *e*′ velocity; TRV, tricuspid regurgitation velocity; PASP, pulmonary artery systolic pressure; TAPSE, tricuspid annular plane systolic excursion; TASA, tricuspid annular systolic acceleration; FAC; fractional area change; LVGLS, global longitudinal strain of left ventricular.

Values shown in bold indicate statistically significant differences (*P* < 0.05) observed when comparing the results across the three groups: non-frail, pre-frail, and frail patients.

### Multivariable regression model

Stepwise forward logistic regression was performed, and the only variable associated with diastolic dysfunction was frailty (OR: 3.49, 95% CI: 1.90–6.39, *P* < 0.001).

## Discussion

The main finding of this study was the significant and independent association between frailty and diastolic dysfunction, with an adjusted OR of 3.49 (95% CI: 1.90–6.39, *P* < 0.001). This result supports the hypothesis that frailty is not only a marker of vulnerability in older adults but may also reflect underlying alterations in cardiac function that predispose individuals to worse outcomes.^23^ Our findings are consistent with previous studies, such as that of Xi *et al.*, who, using a similar multidimensional definition of frailty, identified a higher prevalence of left ventricular hypertrophy and diastolic dysfunction in hospitalized frail patients.^8^

The results of our study show both similarities and differences compared with previous investigations in Anglo-Saxon populations. In the study by Gharacholou *et al.*, conducted at the Mayo Clinic, frailty was associated with increased left atrial volume, lower stroke volume, and higher pulmonary pressures; however, no relationship was identified with left ventricular mass.^12^ Our results confirm the association between frailty and increased left atrial volume, which may reflect chronic pressure overload, but differ in terms of left ventricular mass, as our analysis found a significant difference between frail and non-frail patients. These discrepancies may be explained by differences in the study population, as our analysis focused on hospitalized patients with preserved ejection fraction, whereas Gharacholou’s study included a broader spectrum of ventricular dysfunction.

On the contrary, in the study by Topriceanu *et al.*, based on the 1946 British birth cohort, frailty in middle age was associated with left ventricular hypertrophy, increased filling pressures, and reduced systolic function in older age.^10^ Similarly, our study also found left ventricular hypertrophy when comparing frail and non-frail patients. However, our main finding supports the notion that frailty primarily affects diastolic function. These differences may be explained by the longitudinal design of the British study, which enabled the assessment of long-term cardiac changes, whereas our study evaluated a hospitalized population at a single time point, limiting the ability to analyse progressive alterations in ventricular function.

A notable finding of this study was the significant increase in PASP in frail patients. This increase in PASP is closely associated with the presence of diastolic dysfunction, as one of the diagnostic criteria for diastolic dysfunction is a tricuspid regurgitation velocity >2.8 m/s. Therefore, the elevated PASP further supports the presence of diastolic dysfunction in frail patients, reinforcing its role in this population’s cardiovascular profile.^24^

In contrast to other studies that have reported a reduction in GLS of the left ventricle in frail patients,^8,25^ our analysis did not find significant differences in this parameter between groups. This absence of GLS differences is likely related to the selection criteria of our study, which included only patients with preserved left ventricular function and no structural cardiac damage. By focusing on a population with normal systolic function, the potential impact of frailty on myocardial contractility may have been mitigated, highlighting instead its predominant effect on diastolic function and atrial remodelling.

To avoid confounding, patients with acute heart failure at admission were excluded. Diagnosis was based on the presence of typical symptoms and elevated NT-proBNP levels (as described above), ensuring that echocardiographic findings reflected changes associated with frailty rather than acute decompensation.

We acknowledge that using the New York Heart Association (NYHA) classification to assess functional limitation due to dyspnoea may cause some confusion, as the scale was originally developed specifically for patients with heart failure. However, in our study, the NYHA classification was employed because of its simplicity and widespread use in clinical practice to describe degrees of dyspnoea, irrespective of the underlying aetiology. Therefore, the NYHA classification served as a general measure of functional status rather than as a definitive indicator of heart failure, since patients with heart failure were excluded from the study.

While reverse causality and residual confounding cannot be eliminated in an observational design, the associations observed in our study show that frailty remains independently linked to diastolic dysfunction. There is, however, biological plausibility for a shared, self-reinforcing pathway: low-grade inflammation, neurohormonal activation, and reduced physical activity are known to drive both musculoskeletal catabolism (frailty) and adverse myocardial remodelling (diastolic dysfunction),^26^ potentially creating a vicious cycle in which early frailty and sub-clinical left ventricular dysfunction amplify one another.

Importantly, the prevalence of frailty in our cohort was considerably higher compared with previous studies, with 40.9% of frail patients vs. the 29.1% reported by Xi *et al.*^8^ This suggests that factors such as socioeconomic differences, access to health care, and the management of cardiovascular diseases may influence the prevalence of frailty and its impact on cardiac function.^27–29^

Finally, few studies have evaluated the relationship between frailty and echocardiographic findings, and only one previous study has included Speckle Tracking technology in its analysis. Notably, this is the first study to examine this association in a hospitalized Ibero-American population with preserved ejection fraction, expanding the applicability of these findings and emphasizing the importance of considering frailty as a key determinant in the cardiac assessment of older adults.

## Limitations

The present study has several limitations. First, its observational design allows exploration of associations between frailty and cardiac structure and function, but cannot establish causality. Second, lipid profiles and information on cardiovascular medications were not collected, preventing adjustment for these potential confounders. Although the echocardiography specialist was not informed of the patients’ frailty status, we acknowledge that this did not represent true blinding, as the examiner had direct contact with the patients during image acquisition. Some frailty components were self-reported, introducing possible misclassification bias. Reliance on clinical records may entail data inaccuracies and missing details, and the exclusion of intensive care patients limits applicability to critically ill cohorts. Because only hospitalized patients were evaluated, the findings may not be generalizable to the broader older population. Moreover, although all participants were acutely admitted to internal medicine, cardiology or geriatric wards, the specific reasons for admission or discharge were not recorded, which constitutes an additional potential confounder.

## Conclusions

This study reveals a significant association between frailty and diastolic dysfunction in hospitalized older adults, highlighting the need for specific cardiac assessments in this population.

## Lead author biography


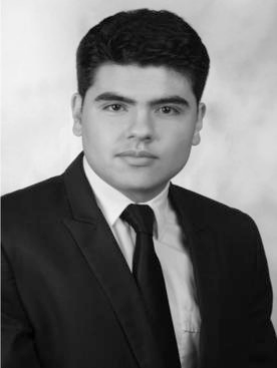


Daniel Betancourt Zuluaga is a medical doctor specializing in Internal Medicine and Geriatrics. He is a member of the Research Group on Geriatrics and Gerontology at the Faculty of Health Sciences, Universidad de Caldas, Colombia. His clinical and academic work focuses on the health and care of older adults, with a particular interest in the intersection of cardiology and geriatrics. He has contributed to scientific publications in respected journals, including the *European Journal of Medical and Health Sciences*. In 2024, he completed an international clinical rotation in cardiogeriatrics at the Hospital Clínico Universitario de Valencia in Spain.

## Supplementary Material

oeaf087_Supplementary_Data

## Data Availability

All dataset analysed are included in this manuscript and supplementary materials.
