# Supplementary material for: Association between frailty and echocardiographic findings in hospitalized older adults with preserved ejection fraction
Source: Eur Heart J Open. 2025 Jul 4;5(4):oeaf087. doi: 10.1093/ehjopen/oeaf087 (PMC12290453; doi:10.1093/ehjopen/oeaf087)
Supplement: oeaf087_Supplementary_Data [file oeaf087_supplementary_data.docx]

**Supplementary table 1.** Baseline characteristics of non-frail, pre-frail, and frail patients, by sex.

|  | **Female** | | | | **Male** | | | ***p*** |
| --- | --- | --- | --- | --- | --- | --- | --- | --- |
|  | **Non-frail**  **(n= 42)** | **Pre-frail**  **(n= 43)** | **Frail**  **(n= 38)** | **Non-frail**  **(n=30)** | | **Pre-frail**  **(n=44)** | **Frail (n=72)** |  |
| Age (y) | 69.0 ± 6.0 | 74.8 ± 7.7 | 76.9 ± 9.0 | 67.6 ± 6.4 | | 75.3± 8.9 | 75.3± 8.9 | 0.638* |
| BMI (kg/m^2^) | 25.9 ± 3.8 | 25.0 ± 4.1 | 24.6 ± 3.6 | 26.3 ± 3.6 | | 24.8 ± 4.0 | 24.9 ± 5.1 | 0.810* |
| Arterial hypertension | 24 (57.1%) | 26 (60.7%) | 25 (65.8%) | 19 (63.3%) | | 34 (77.3%) | 51 (70.8%) | 0.099† |
| Diabetes mellitus | 5 (11.9%) | 15 (34.9%) | 10 (26.3%) | 6 (20.0%) | | 15 (34.1%) | 19 (26.4%) | 0.674† |
| Atrial fibrillation | 1 (2.4%) | 3 (7.0%) | 2 (5.3%) | 2 (6.7%) | | 2 (4.6%) | 7 (9.7%) | 0.455‡ |
| CKD | 5 (11.9%) | 4 (9.3%) | 4 (10.5%) | 2 (6.7%) | | 4 (9.1%) | 7 (9.7%) | 0.682‡ |
| COPD | 1 (2.4%) | 9 (20.0%) | 13 (34.2%) | 2 (6.7%) | | 7 (15.9%) | 26 (36.1%) | 0.302‡ |
| OSA | 2 (4.8%) | 0 (0.0%) | 2 (5.3%) | 1 (3.3%) | | 1 (2.3%) | 2 (2.8%) | 1.000‡ |
| Coronary heart disease | 6 (14.3%) | 6 (14.0%) | 4 (10.5%) | 3 (10.0%) | | 4 (9.1%) | 5 (6.9%) | 0.231‡ |
| Smoking history | 3 (7.1%) | 2 (4.7%) | 4 (10.5%) | 2 (6.7%) | | 3 (6.8%) | 4 (5.6%) | 0.808‡ |
| NYHA ≥III | 2 (4.8%) | 6 (14.0%) | 21 (55.3%) | 1 (3.3%) | | 7 (15.9%) | 32 (44.4%) | 0.487‡ |

BMI: body mass index; CKD: chronic kidney disease; COPD: chronic obstructive pulmonary disease; OSA: obstructive sleep apnea; NYHA: New York Heart Association functional class.

*Two-way mixed ANOVA

†Chi-square test
‡Fisher's exact test

**Supplementary table 2.** Cardiac structural parameters of non-frail, pre-frail, and frail patients, by sex.

|  | **Female** | | | | **Male** | | | ***p*** |
| --- | --- | --- | --- | --- | --- | --- | --- | --- |
|  | **Non-frail**  **(n= 42)** | **Pre-frail**  **(n= 43)** | **Frail**  **(n= 38)** | **Non-frail**  **(n=30)** | | **Pre-frail**  **(n=44)** | **Frail (n=72)** |  |
| LVESD (mm) | 26.2 ± 4.2 | 26.5 ± 4.7 | 26.0 ± 5.3 | 24.9 ± 3.6 | | 25.6 ± 4.9 | 24.5 ± 4.3 | **0.024*** |
| LVEDD (mm) | 43.8 ± 4.2 | 43.4 ± 4.8 | 42.8 ± 8.2 | 42.6 ± 3.4 | | 40.5 ± 4.3 | 40.5 ± 4.9 | **0.001*** |
| IVS (mm) | 8.5 ± 1.3 | 8.7 ± 1.6 | 9.3 ± 1.7 | 8.0 ± 1.0 | | 8.2 ± 1.2 | 8.6 ± 1.4 | **0.002*** |
| LVPW (mm) | 8.3 ± 1.3 | 8.4 ± 1.3 | 9.3 ± 2.5 | 7.9 ± 0.9 | | 8.0 ± 1.0 | 8.3 ± 1.1 | **<0.001*** |
| LVEDV (ml/m^2^) | 56.2 ± 11.3 | 58.0 ± 8.3 | 57.8 ± 15.1 | 52.4 ± 8.7 | | 53.3 ± 10.1 | 50.9 ± 10.5 | **<0.001*** |
| LVESV (ml/m^2^) | 24.3 ± 7.8 | 24.4 ± 4.7 | 25.1 ± 6.5 | 21.0 ± 4.2 | | 21.5 ± 4.9 | 20.1 ± 4.7 | **<0.001*** |
| RWT | 0.39 ± 0.06 | 0.40 ± 0.08 | 0.43 ± 0.07 | 0.38 ± 0.05 | | 0.39 ± 0.06 | 0.42 ± 0.07 | 0.155* |
| LVMI (g/m^2^) | 63.2 ± 15.5 | 67.5 ± 14.7 | 78.2 ± 23.3 | 62.9 ± 11.8 | | 64.5 ± 14.3 | 70.2 ± 21.1 | 0.063* |
| RVD_basal_ (mm) | 37.7 ± 4.1 | 39.1 ± 4.6 | 34.1 ± 5.0 | 33.0 ± 4.5 | | 34.1 ± 5.0 | 35.4 ± 5.4 | **<0.001*** |
| RVD_mid_ (mm) | 33.5 ± 3.5 | 34.0 ± 3.9 | 34.7 ± 4.9 | 29.0 ± 4.4 | | 30.1 ± 4.3 | 31.3 ± 4.7 | **<0.001*** |
| RVD_long_ (mm) | 71.5 ± 6.7 | 70.9 ± 5.8 | 71.4 ± 7.7 | 65.3 ± 6.9 | | 64.2 ± 7.6 | 65.7 ± 6.5 | **<0.001*** |
| LAD (mm) | 40.3 ± 5.3 | 39.7 ± 9.8 | 41.1 ± 6.6 | 37.6 ± 4.0 | | 39.3 ± 5.1 | 40.5 ± 7.9 | 0.189* |
| LAVI (ml/m^2^) | 31.1 ± 8.1 | 35.1 ± 8.4 | 37.5 ± 10.8 | 29.7 ± 7.9 | | 34.9 ± 9.4 | 40.7 ± 14.4 | 0.567* |
| RAVI (ml/m^2^) | 22.9 ± 7.8 | 25.1 ± 7.0 | 26.5 ± 8.5 | 19.5 ± 4.3 | | 22.6 ± 6.1 | 25.7 ± 9.5 | **0.035*** |

*LVESD* left ventricular end systolic diameter, *LVEDD* left ventricular end diastolic diameter, *IVS* interventricular septum, *LVPW* left ventricular posterior wall, *LVEDV* left ventricular end diastolic volume, *LVESV* left ventricular end systolic volume, *RWT* relative wall thickness, *LVMI* left ventricular mass index, *RVD_basal_* right ventricular basal diameter, *RVD*_mid_ right ventricular mid diameter, *RVL_long_* right ventricular longitudinal diameter, *LAD* left atrial diameter, *LAVI* left atrial volume index, *RAVI* right atrial volume index.

* Two-way mixed ANOVA

*LVMI non-frail vs frail* P: 0.000 (Student`s t-test).

**Supplementary table 3.** Cardiac functional parameters of non-frail, pre-frail, and frail patients, by sex.

|  | **Female** | | | | **Male** | | | ***p*** |
| --- | --- | --- | --- | --- | --- | --- | --- | --- |
|  | **Non-frail**  **(n= 42)** | **Pre-frail**  **(n= 43)** | **Frail**  **(n= 38)** | **Non-frail**  **(n=30)** | | **Pre-frail**  **(n=44)** | **Frail**  **(n=72)** |  |
| LVEF(%) | 59.0 ± 3.6 | 57.7 ± 5.2 | 58.4 ± 3.8 | 60.5 ± 3.8 | | 59.9 ± 3.8 | 60.5 ± 3.9 | **<0.001*** |
| E/A | 0.81 ± 0.26 | 0.80 ± 0.21 | 0.66 ± 0.13 | 0.86 ± 0.22 | | 0.80 ± 0.31 | 0.83 ± 0.33 | **0.027*** |
| E/e’ | 8.4 ± 2.5 | 9.9 ± 3.0 | 10.6 ± 3.6 | 9.1 ± 2.2 | | 11.3 ± 4.1 | 13.3 ± 7.2 | **0.006*** |
| Se’V (cm/s) | 6.3 ± 1.5 | 8.4 ± 1.3 | 9.3 ± 2.5 | 7.9 ± 0.9 | | 8.0 ± 1.0 | 8.3 ± 1.1 | 0.892* |
| Le’V (cm/s) | 8.0 ± 1.7 | 7.3 ± 2.2 | 6.4 ± 2.3 | 7.8 ± 1.8 | | 6.8 ± 2.0 | 6.6 ± 1.7 | **<0.001*** |
| TRV (m/s) | 2.7 ± 0.4 | 2.8 ± 0.2 | 2.9 ± 0.3 | 2.6 ± 1.3 | | 2.9 ± 0.4 | 2.9 ± 0.5 | 0.651* |
| PASP (mmHg) | 38.8 ± 14.2 | 36.8 ± 12.7 | 40.3 ± 10.3 | 34.8 ± 4.4 | | 41.8 ± 12.8 | 41.3 ± 11.5 | 0.593* |
| TAPSE (mm) | 22.4 ± 2.6 | 22.2 ± 3.1 | 22.9 ± 3.2 | 20.8 ± 2.2 | | 20.9 ± 3.2 | 21.1 ± 4.0 | **<0.001*** |
| TASA (cm/s) | 12.7 ± 2.1 | 12.8 ± 2.3 | 13.3 ± 3.8 | 11.5 ± 1.8 | | 12.6 ± 3.0 | 11.8 ± 2.2 | **0.004*** |
| FAC (%) | 47.7 ± 6.1 | 47.7 ± 5.7 | 46.9 ± 5.7 | 48.9 ± 6.1 | | 49.6 ± 7.9 | 48.6 ± 5.9 | **0.043*** |
| LVGLS (%) | -23.1 ± 2.0 | -21.6 ± 2.0 | -21.5 ± 3.3 | -22.9 ± 2.2 | | -22.4 ± 3.0 | -21.5 ± 3.3 | 0.193* |

*LVEF left ventricular ejection fraction, Se’V septal e’ velocity, Le’V lateral e’ velocity, TRV tricuspid regurgitation velocity, PASP pulmonary artery systolic pressure, TAPSE tricuspid annular plane systolic excursion, TASA tricuspid annular systolic acceleration, FAC fractional area change, LVGLS global longitudinal strain of left ventricular.*

*Two-way mixed ANOVA

**Supplementary table 4.** Distribution of Fried Frailty Index components among non-frail, pre-frail, and frail patients.

|  | **Non-frail**  **(n= 72)** | **Pre-frail**  **(n= 87)** | **Frail**  **(n= 110)** | **Total**  **(n= 269)** |
| --- | --- | --- | --- | --- |
| Unintentional weight loss | 0 (0.0%) | 13 (14.9%) | 50 (45.4%) | 63 (23.4%) |
| Self-reported exhaustion | 2 (2.7%) | 9 (10.3%) | 63 (57.3%) | 74 (27.5%) |
| Weakness (grip strenght) | 29 (40.2%) | 49 (56.3%) | 88 (80.0%) | 166 (61.7%) |
| Slow walking speed | 65 (90.2%) | 73 (83.9%) | 97 (88.2%) | 235 (87.4%) |
| Low physical activity | 22 (30.5%) | 61 (70.1%) | 109 (99.1%) | 192 (71.3%) |

**Supplementary table 5.** Distribution of diastolic dysfunction criteria among non-frail, pre-frail, and frail patients.

|  | **Non-frail**  **(n= 72)** | **Pre-frail**  **(n= 87)** | **Frail**  **(n= 110)** | **Total**  **(n= 269)** |
| --- | --- | --- | --- | --- |
| E/e’ >14 | 0 (0.0%) | 12 (14.8%) | 25 (24.0%) | 37 (14.6%) |
| Se’V <7 cm/s or Le’V <10 cm/s | 41 (59.4%) | 58 (71.6%) | 89 (85.6%) | 188 (74.0%) |
| TRV >2.8 m/s | 3 (4.1%) | 19 (21.8%) | 35 (31.8%) | 57 (21.2%) |
| LAVI >34 ml/m^2^ | 17 (23.6%) | 43 (49.4%) | 69 (62.7%) | 129 (48.0%) |
| Patients with >50% of criteria | 15 (25.4%) | 19 (32.2%) | 25 (42.4%) | 59 (21.9%) |

*Se’V septal e’ velocity, Le’V lateral e’ velocity, TRV tricuspid regurgitation velocity, LAVI* left atrial volume index.
